# Effectiveness of Mobile Exergaming with Sensor-Based Visual Feedback as an Adjunct Therapy for Home-Based Quadriceps Exercise Training in Knee Osteoarthritis: A Prospective Randomized Controlled Trial

**DOI:** 10.3390/life15111738

**Published:** 2025-11-12

**Authors:** Chavarat Jarungvittayakon, Paphon Sa-ngasoongsong, Danai Chockchaisakul, Jaturong Bamrungchaowkasem, Siwadol Wongsak

**Affiliations:** Department of Orthopedics, Faculty of Medicine Ramathibodi Hospital, Mahidol University, Bangkok 10400, Thailand; chavarat.jar@mahidol.ac.th (C.J.); paphon.sag@mahidol.ac.th (P.S.-n.); danai.ccs@gmail.com (D.C.); jaturongliv@gmail.com (J.B.)

**Keywords:** IMU sensor, mobile application, knee rehabilitation, quadriceps muscle power, range of motion

## Abstract

Background: Exergame applications were introduced recently for orthopedic rehabilitation. This study aimed to evaluate the effectiveness of a 6-week home-based quadriceps exercise with mobile exergaming for treatment of primary knee osteoarthritis (KOA). Methods: A prospective randomized controlled trial was conducted in 56 primary KOA patients. All patients were allocated into two groups (*n* = 28 each group). Intervention group received the 6-week mobile exergaming program using a smartphone exercise game with a wearable wireless motion sensor. Control group received a standard 6-week exercise program. Outcomes were active knee arc of motion, quadriceps muscle power, visual analog scale score, timed “Up and Go” (TUG) test, and Knee Injury and Osteoarthritis Outcome Score (KOOS) at a 6-week follow-up. Results: At 6-week post-exercise, the intervention group significantly improved quadriceps power, arc of motion, VAS score at rest and on motion, TUG test, and KOOS-symptom domain compared to the control group (*p* < 0.05 all). No device- or exercise-related complications were found. Conclusions: Mobile exergaming with visual feedback control using a wearable wireless sensor significantly improves KOA outcomes compared to standard home-based exercise as early as 6 weeks post-application.

## 1. Introduction

Knee osteoarthritis (KOA) is a common cause of chronic knee pain that leads to disability in elderly patients. KOA can be associated with symptoms of pain, reduction in quadriceps strength and range of motion, and consequently, deterioration in quality of life and function. This functional limitation results in an increase in the risk of morbidity and mortality [1,2]. Regarding the non-pharmacological treatment for KOA, strengthening and range-of-motion exercises are among the first-line recommendations for managing KOA [3] and have been generally used for alleviating the symptoms, enhancing muscle strength, and improving patients’ physical function and quality of life [4,5,6,7]. However, the outcomes after these exercises are highly variable due to poor patient compliance and adherence and the need for requiring exercise promotion and encouragement to maximize the impacts/benefits of life-long exercise [8,9]. Recent research showed that the key factors relevant to increasing the adherence to physical exercise regimens depends on several aspects, such as the characteristics of the exercise program, supervision, technology, enjoyment and absence of unpleasant experiences, communication and feedback, and availability for progression and monitoring [10]. Collectively, these aspects make developing an effective exercise intervention in patients with KOA challenging.

Over the last few decades, “exergame” (or exergaming)—defined as the use of game design elements to assist the exercise intervention by using game mechanics (quests, points, leaderboards, badges) in an effort to improve exercise engagement and treatment outcomes [11,12]—has become an effective approach for advancing health promotion and disease prevention in the individuals at risk of impairment in cognitive, physical, functional, and emotional well-being. The exergame applications for knee joint rehabilitation can be used through an active video game [13,14], smartphone [15], or virtual reality game [16]. Moreover, due to the recent advancement of sensor technology, the application of a wearable knee sensor has enabled us to measure and monitor the knee range of motion during exercise and then send feedback to the patient, resulting in better patient compliance and improved knee outcomes [17]. To our knowledge, only a few studies have reported the efficacy of the combined use of exergaming and a wearable knee sensor for knee rehabilitation [18,19,20]. However, none of the previous studies has explored the effectiveness of this strategy in patients with KOA. The current study therefore aimed to evaluate the effectiveness of innovative mobile exergaming with a wearable knee sensor device on the 6-week home-based quadriceps strengthening exercise (QSE) training for KOA in terms of pain, range of motion, quadriceps muscle power, and knee-related physical function compared to the standard exercise training in patients diagnosed with primary mild-to-moderate KOA.

## 2. Materials and Methods

### 2.1. Study Design, Inclusion, and Exclusion Criteria

This study was designed as a prospective randomized controlled trial with a 1:1 allocation ratio. Prior approval from the Human Research Ethics Committee, Faculty of Medicine Ramathibodi Hospital, Mahidol University, was obtained (Protocol No. 09-61-13, COA. No. MURA2018/655), and the clinical trial was successfully registered (Trial Registration No. TCTR20230130005). Informed consent was obtained from all patients who participated in this study before the treatment allocation, in accordance with the Declaration of Helsinki. The manuscript was prepared according to the Consolidated Standards of Reporting Trials 2025 guidelines [21].

The study included patients who (1) were diagnosed with primary KOA according to the American College of Rheumatology criteria [22] with Kellgren and Lawrence [23] stage 2–3 [24] and scheduled for conservative treatment, (2) were aged between 45 and 75 years, (3) had WHO functional class 1, and (4) could use a mobile device and wearable knee sensors. Exclusion criteria included patients who (1) had previous significant injury or surgery, (2) had a previous intra-articular injection as a KOA treatment within past 6 months, (3) had neuromuscular diseases (e.g., stroke), (4) had a history of inflammatory joint diseases (e.g., rheumatoid arthritis or gout), (5) had significant chronic illnesses (e.g., end-stage renal disease or liver cirrhosis), (6) were contraindicated for wearable knee devices (e.g., due to skin allergy to the sensor device).

**Figure 1:**
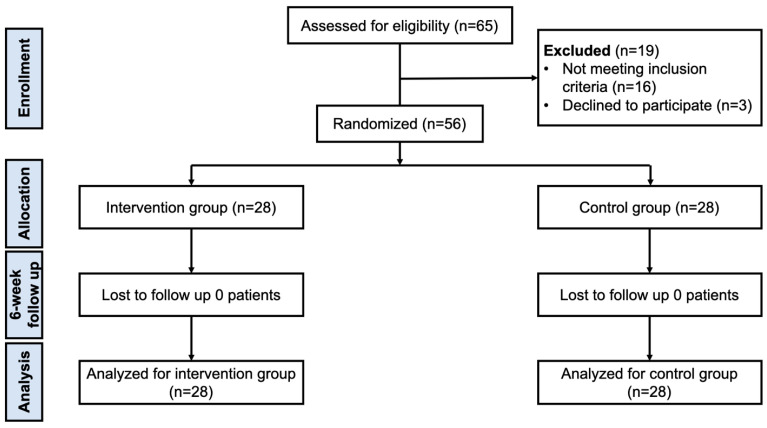
Flow diagram of this study.

All patients were randomized into 2 groups (intervention group or control group) using computer-generated blocked randomization with various block sizes through STATA version 14.0 (StataCorp, College Station, TX, USA). The randomization was then concealed into the sequentially numbered, opaque, sealed envelopes. The envelopes containing treatment assignments were opened sequentially by a research assistant who was not involved in the concealment process after recruitment. After enrollment, the patients were randomly assigned to either the intervention group or control group (Figure 1). All patients received the same standard conservative treatment, including the quadriceps strengthening exercise (5 repetitions of deep knee flexion and full knee extension, twice a day), concomitant KOA medications, as well as weight reduction and self-care advice. In the control group, the quadriceps strengthening exercise was instructed by a research assistant on the first day after randomization, and the patients were asked to repeat the information and demonstrate one set of exercise for confirmation of understanding (teach-back method) [25]. The adherence on the exercise program was routinely checked with a daily phone call or via a communication app (LINE app, version 13.20.0). In the intervention group, patients were instructed and trained to use a mobile application device and sensors by a research assistant who was not involved in the data collection and analysis. Patients were then evaluated for the proper use of the device before starting the study.

### 2.2. Information Related to Mobile Exergaming Device and Quadriceps Exercise Protocol

**Figure 2:**
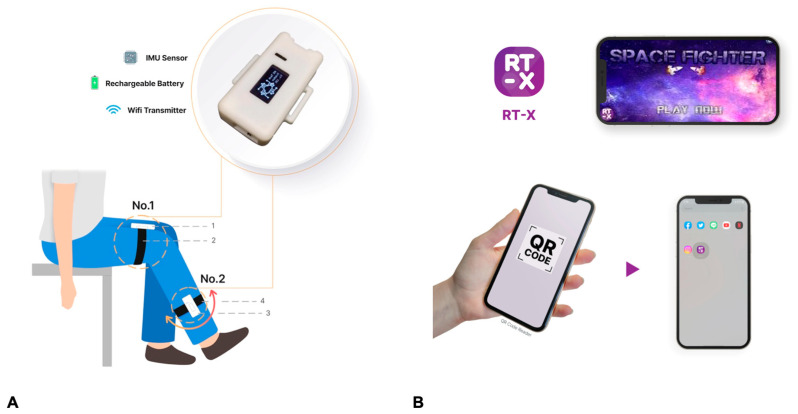
The (**A**) application of knee IMU sensor (the first sensor [No.1] at 10 cm above the knee joint on the anterior thigh and the second sensor [No.2] at 10 cm below the knee joint on the lateral calf) and (**B**) installation of mobile application.

Our mobile exergaming device comprised a wearable sensor instrument and a mobile game. The sensor instrument consisted of two plastic boxes sized at 4 × 3 × 1 cm containing an IMU sensor, internal chargeable battery, and a Wi-Fi transmitter. The first plastic box was attached 10 cm above the knee at the anterior thigh, and the other was attached 10 cm below the knee at the lateral calf (Figure 2). Both boxes are connected to each other via a Wi-Fi hotspot and can measure the relative angle between them using IMU sensors. The first device measures the angle relative to the ground, while the second device measures the angle relative to the Earth’s vertical axis, based on their respective positions. The program then calculates the angular difference between the two devices to display the knee flexion–extension angle. The sensors connect to a smartphone through the same Wi-Fi hotspot, and the game-based program is developed as a mobile application compatible with both iOS and Android systems. All data, including usage time and knee flexion–extension angles, are automatically synchronized with an online cloud database for patient data storage.

**Figure 3:**
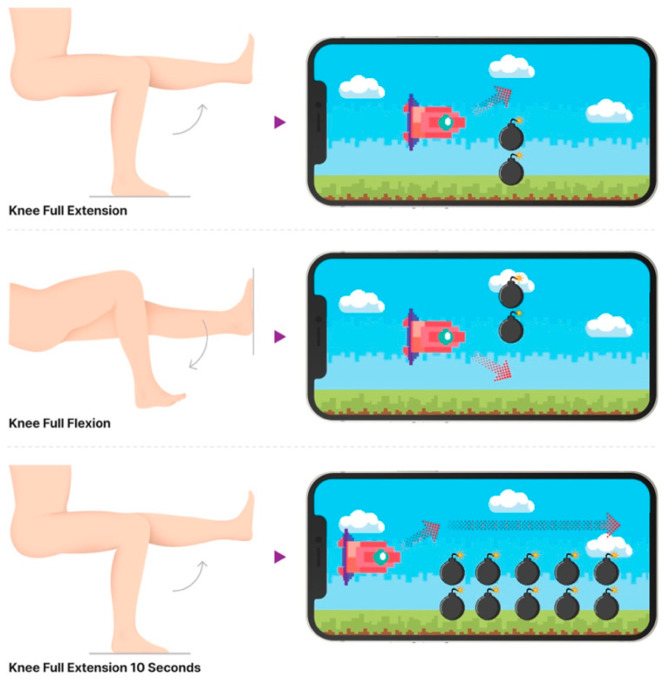
Demonstration of the use of mobile exergaming with app-based visual feedback for active range-of-motion knee exercise.

The mobile game was developed as a flight pilot simulation game app for encouraging patients to exercise (Figure 2). In the game, moving the airplane up or down to pass obstacles required the patients to perform deep knee flexion and full extension. The game was designed for patients to perform 5 repetitions of deep knee bending and full knee stretching while maintaining the full knee extension position for 10 s each time (Figure 3). Patients had to complete the game twice a day: the first set between 00.01 and 12.00 am and the second set between 00.01 and 12.00 pm. If a patient did not perform the exercise, the app notified the research assistant through a cell phone; the researcher would then call to remind the patient to complete the exercise protocol. Data related to the patient range of motion while using the device was recorded by X and collected for analysis. Before the application, each device was calibrated with the standard measurement tools by National Institute of Metrology Thailand (certificate of calibration before DA-0023-19).

### 2.3. Data Collection

Baseline characteristics—including age, gender, body weight, height, and BMI—were collected. The data related to KOA for the 10-point visual analog scale score [26] at rest and during knee motion, arch of active knee range of motion, quadriceps muscle power, TUG test, and KOOS [27], were collected before and after the 6-week exercise program at the outpatient clinic by one of the authors (CJ). All patients were asked to rate the level of satisfaction at the end at 6-week exercise program using 5-point Likert scale (1 = very dissatisfied, 2 = dissatisfied, 3 = neutral, 4 = satisfied, and 5 = very satisfied).

The range of motion was evaluated by the sensor device, and the quadriceps muscle power was measured by a digital hand-held dynamometer (HHD) (MicroFET2™, Hoggan Health Industries, Draper, UT, USA) with a standard knee extensor measurement protocol (measuring patients in the sitting position with full thigh support and 30° knee flexion, applying the HHD at the distal third of the tibia, and instructing the patients to progressively increase their effort to the maximal quadriceps strength level for at least 5 s) [28].

### 2.4. Statistical Analysis

Statistical analyses were performed using STATA software. Intention to treat analysis was applied. Continuous data with non-normalized and normalized distribution were reported as mean ± standard deviation and median (range). Paired *t*-test and Wilcoxon test were used to compare the differences between the time before the study and at 6 weeks within the same group. Unpaired *t*-test and Mann–Whitney test were used to compare the groups. Categorical data were expressed in percentages, and Fisher’s exact test was used to compare differences between both groups. Statistical significance was determined as *p*-value < 0.05.

### 2.5. Sample Size Calculation

The sample size estimation was calculated from data from a pilot study in 20 KOA patients in our hospital, using the VAS score on motion. The mean ± SD of 10 mm VAS was 5.0 ± 1.2 mm. The following parameters were applied: alpha error (α) = 0.05, power of the study (1 − ß) = 0.8, difference in population means (δ) as minimally clinical important difference = 1.0 mm [29], and ratio between groups = 1:1. Therefore, the sample size required each group to include 23 participants. After adding the 20% drop-out rate (*n* = 5), the final sample size was a total of 56 participants (28 per group).

## 3. Results

### 3.1. Patients’ Characteristic Data

Between January 2019 and January 2020, a total of 56 patients with KOA were recruited into this study (Figure 1). Table 1 shows the patients’ demographic data. No significant difference was found in patients’ baseline characteristics or the pre-exercise data between both groups (*p* > 0.05 all). All patients completed the follow-up visits.

### 3.2. Outcomes of This Study

**Figure 4:**
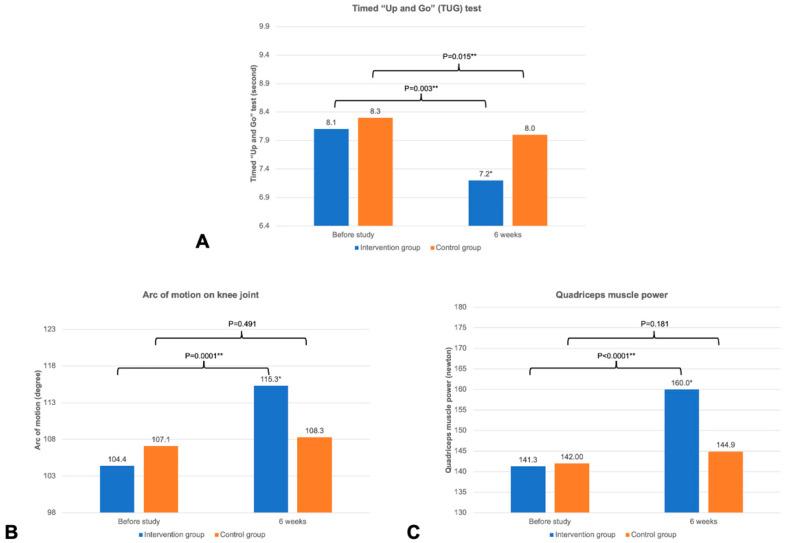
Changes in knee joint outcomes. (**A**) Timed “Up and Go” (TUG) test, (**B**) arc of motion of the knee joint, and (**C**) quadriceps muscle power. * Significant difference between intervention and control group with *p* < 0.05; ** significant difference between the time before the study and after 6 weeks within the same group, with *p* < 0.05.

**Figure 5:**
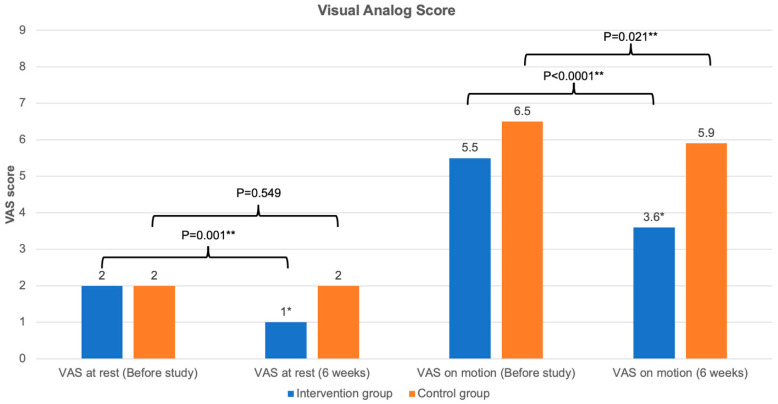
Changes in visual analog score. * Significant difference between intervention and control group with *p* < 0.05; ** significant difference between the time before the study and after 6 weeks within the same group, with *p* < 0.05.

Figure 4 and Figure 5 illustrate the changes in the TUG test, arc of knee motion, quadriceps muscle power, and VAS score between the baseline and 6-week follow-up. Table 2 shows the comparison of the outcomes of the knee exercise between the groups at 6 weeks. Regarding the changes at 6 weeks after the study, the intervention group completing the mobile app-based feedback exercise showed a significant improvement in all knee exercise outcomes, including the TUG test, knee flexion and extension, arc of motion, quadriceps power, VAS score at rest, and VAS score on motion (*p* < 0.05 all). However, the control group following the standard knee exercise program demonstrated a significant improvement only in the TUG test and VAS score on motion (*p* < 0.05 both) (Figure 4 and Figure 5).

**Figure 6:**
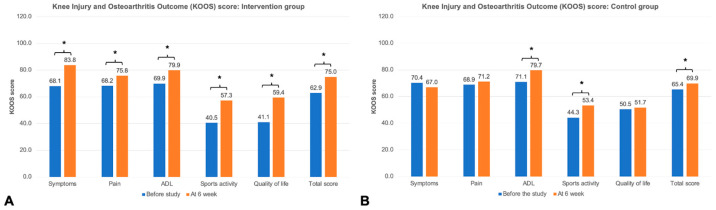
Changes in each domain of Knee Injury and Osteoarthritis Outcome Score (KOOS). (**A**) KOOS in the intervention group, (**B**) KOOS in the control group. * Significant difference between the time before the study and at 6 weeks, with *p* < 0.05.

Figure 6 shows the changes in KOOS between before the study and at 6 weeks for both groups. The intervention group demonstrated significant improvement in all KOOS subdomains (*p* < 0.05 all) (Figure 6A), whereas the control group demonstrated significant improvement only in the activity of daily living (ADL), sports activity, and total score (*p* < 0.05 all) (Figure 6B).

At the 6-week follow-up, the intervention group had significantly improved in all knee exercise outcomes—including the TUG test, arc of motion, quadriceps power, VAS score at rest, and VAS score on motion—compared to the control group (*p* < 0.05 all) (Figure 4 and Figure 5). However, the knee functional assessment, as measured by the KOOS, in the intervention group did not show a significant difference compared to the control group, except for the symptom domain (83.8 ± 10.4 in the intervention group vs. 67.0 ± 17.4 in the control group, *p* < 0.0001) (Table 2). The intervention group also demonstrated significantly higher satisfaction score at the end of the study compared to the control group (*p* < 0.0001).

## 4. Discussion

QSE is one of the most common types of training exercises for treating KOA [7] and is recommended as the first-line management for KOA due to the efficacy related to significant knee pain reduction and physical function enhancement [3,22,30]. However, the effectiveness of QSE is highly variable and significantly depends on individual patient’s engagement and compliance [31]. This study therefore aimed to evaluate the usefulness of mobile exergaming with sensor-based visual feedback for assisting with 6-week home-based QSE in terms of pain, quadriceps power, and KOA-related functional outcomes.

The results from the present study showed that both intervention and control groups experienced significant improvement in KOA-related outcomes (Figure 4, Figure 5 and Figure 6) as compared to previous studies on QSE [26,32]. After a standard 6-week home-based QSE in the control group, data showed a significant improvement only in some KOA outcomes, such as the TUG test, VAS score on motion, and some KOOS subdomains (ADL, sports activity, and global score). However, the intervention group, who used adjunct therapy with mobile exergaming with feedback control through a wearable knee sensor, significantly improved all KOA outcomes, including pain (VAS scores at rest and on motion), knee joint stiffness (arc of motion), quadriceps muscle power, physical function (TUG test), and overall knee score (KOOS) (*p* < 0.05 all). These results are comparable to those in a previous study using exergame for KOA [13,16,33]. Moreover, the intervention group also showed a statistically significantly better in knee flexion and extension, arc of motion, quadriceps muscle power, VAS at rest and on motion, KOOS symptoms domain, and satisfaction score, compared to the control group (*p* < 0.05 all). The significant improvement in KOOS symptoms domain might be explained by the significant improvement in arc of motion and stiffness which is directly related to this domain. Therefore, the findings related to the effectiveness of mobile exergaming with a wearable knee sensor in this study could be explained by the effect of gamification with mobile exergaming as a guided physiotherapy, the real-time visual feedback control via a mobile app and a wearable knee sensor, and the advantages of remote monitoring through an internet system. First, the adjunct gamification with the QSE improved the exercise engagement as an additional incentive for rehabilitation and reduced the psychological barriers to exercising for patients with KOA [18]. Second, the real-time feedback control through a mobile app with a wearable knee sensor helped patients perform an accurate and better knee-joint motion exercise with deep knee flexion and full knee extension during the QSE [34,35]. Third, the remote monitoring through the internet helped remind patients who did not comply with the daily program, thereby improving the patients’ compliance during the 6-week training [36].

This study also has some limitations. First, although this study was a prospective randomized controlled trial, the sample size was relatively small, and follow-up time was only 6 weeks. However, a previous study by Lin et al. showed that the effectiveness of exergaming using an active video game did not significantly differ after the intervention ended [13]. Future clinical trials with a larger sample size and a long-term follow-up are therefore needed to further evaluate the effectiveness of this strategy on patient compliance and adherence [35]. Moreover, the application of mobile exergaming in some elderly patients may be limited due to the lack of familiarity in modern technology and required further development [37]. Second, the wearable knee sensor in this study was designed as a simple system with two IMU sensors applied on the thigh and at the mid-leg level. This design was used because our study focuses only on the QSE in the sitting position; a previous study by Franco et al. demonstrated excellent accuracy of knee angle recognition with an average error of 0.6 degree [38]. Due to those differences, the efficacy of the current design might not be directly comparable with other designs using multiple sensors on the lower back, thigh, leg, and foot, which focused on the assessment of walking or completing ADLs [17].

## 5. Conclusions

Our study supports the use of innovative technology (smartphone and wearable knee sensor) as an adjunct for improving the outcome of non-operative treatment in KOA. The results from this study showed that mobile exergaming with sensor-based visual feedback through a wearable knee sensor could significantly reduce knee pain at rest and during motion, increase quadriceps muscle power and arc of motion, and improve the physical function and functional score in patients with KOA compared to the standard QSE program.

## 6. Patents

The innovative device and related software in this study are under petty patent and copyright registrations in Thailand (application number 2003002068).

## Figures and Tables

**Table 1:** Demographic data of both groups in this study.

	Intervention Group (*n* = 28)	Control Group (*n* = 28)	*p*-Value
Age, year ^a^	59.9 (5.8)	62.1 (5.6)	0.158
Male–female ^b^	5:23	6:22	1.000
BMI, kg/m^2 a^	26.1 (3.2)	27.1 (3.6)	0.268
Extension, degree ^a^	10.9 (6.2)	9.0 (4.5)	0.205
Flexion, degree ^a^	115.6 (12.2)	116.4 (10.1)	0.803
Arc of motion, degree ^a^	104.8 (15.6)	107.4 (10.2)	0.463
Quadriceps muscle power, N ^a^	141.3 (23.2)	142.0 (19.2)	0.911
VAS score at rest ^c^	2 (0–6)	2 (0–5)	0.620
VAS score on motion ^a^	5.5 (2.0)	6.5 (2.4)	0.125
TUG test, second ^a^	8.1 (1.5)	8.3 (2.6)	0.753
KOOS ^a^			
Symptoms	68.1 (21.0)	70.4 (16.4)	0.652
Pain	68.2 (17.3)	68.9 (19.9)	0.887
ADL	69.9 (16.9)	71.1 (18.0)	0.790
Sports activity	40.5 (21.5)	44.3 (24.0)	0.541
Quality of life	41.0 (20.0)	50.5 (19.4)	0.081
Total score	63.0 (16.9)	65.4 (17.2)	0.604

BMI, body mass index; VAS, visual analog scale; TUG, timed “Up and Go”; KOOS, Knee Injury and Osteoarthritis Outcome Score; ADL, activities of daily living. ^a^ value presented as mean (standard deviation); ^b^ value presented as ratio; ^c^ value presented as median (range).

**Table 2:** Results at the 6-week follow-up period.

	Intervention Group (*n* = 28)	Control Group (*n* = 28)	*p*-Value
Extension, degree ^a^	7.8 (5.7)	5.6 (3.6)	0.94
Flexion, degree ^a^	123.1 (8.4)	114.4 (8.0)	0.0002 *
Arc of motion, degree ^a^	115.3 (10.6)	108.8 (7.9)	0.011 *
Quadriceps muscle power, N ^a^	160.0 (24.1)	144.9 (15.6)	0.007 *
VAS score at rest ^b^	1 (0–5)	2 (0–5)	0.025 *
VAS score on motion ^a^	3.6 (2.1)	5.9 (2.2)	0.0002 *
TUG test, second ^a^	7.2 (1.4)	8.0 (2.3)	0.145
KOOS ^a^			
Symptoms	83.8 (10.4)	67.0 (17.4)	<0.0001 *
Pain	75.8 (21.0)	71.2 (17.8)	0.379
ADL	79.9 (15.5)	79.7 (13.6)	0.964
Sports activity	57.3 (27.8)	53.4 (20.6)	0.551
Quality of life	59.4 (28.6)	51.7 (25.5)	0.290
Total score	75.0 (16.5)	69.9 (14.4)	0.227
Satisfaction score ^a^	4.6 (0.5)	3.4 (0.6)	<0.0001 *

VAS, visual analog scale; TUG, timed “Up and Go”; KOOS, Knee Injury and Osteoarthritis Outcome Score; ADL, activities of daily living. ^a^ value presented as mean (standard deviation); ^b^ value presented as median (range). * Significant difference between intervention and control group with *p*-value < 0.05.

## Data Availability

The raw data supporting the conclusions of this article will be made available by the authors on request.

## Abbreviations

The following abbreviations are used in this manuscript:

KOA	Knee osteoarthritis
VAS	Visual analog scale score
TUG	Timed “Up and Go”
KOOS	Knee Injury and Osteoarthritis Outcome Score
QSE	Quadriceps strengthening exercise
KL	Kellgren and Lawrence
HHD	Hand-held dynamometer
